# Clinical efficacy of curcumin in treating oral submucous fibrosis and dynamic analysis of associated cytokines

**DOI:** 10.1097/MD.0000000000045423

**Published:** 2026-01-02

**Authors:** Juanhua Chai, Yang Li, Lu Zhang, Mengfei Yao, Yuxin Gu, Liye Song

**Affiliations:** a Department of Stomatology, Affiliated Hospital of Hebei University of Engineering, Handan, Hebei, China; b Department of Stomatology, Renqiu People’s Hospital, Renqiu, Hebei, China.

**Keywords:** curcumin, inflammatory cytokines, oral submucous fibrosis, retrospective cohort study

## Abstract

Oral submucous fibrosis (OSF) is a chronic, inflammation-driven precancerous condition. Current corticosteroid treatments often yield short-lived effects with adverse reactions. Curcumin’s anti-inflammatory, antifibrotic, and immunomodulatory properties suggest therapeutic potential, but clinical evidence remains limited. This retrospective study enrolled 110 OSF patients (June 2023–September 2024), divided into a curcumin group (*n* = 54) and a triamcinolone group (*n* = 56), treated for 4 weeks. Outcomes included clinical efficacy, symptom improvement (visual analog scale [VAS] score, mouth opening, mucosal lesion area), and cytokine levels (IFN-γ, TGF-β1, TNF-α). The curcumin group showed higher total efficacy (88.89% vs 73.21%, *P* = .037), faster pain reduction (VAS: 2.25 ± 0.52 vs 2.86 ± 0.43 at week 4, *P* < .001), greater mouth opening (36.88 ± 2.07 mm vs 35.67 ± 2.71 mm, *P* < .05), and reduced lesion area (4.63 ± 0.54 cm² vs 5.61 ± 0.69 cm², *P* < .001). Serologically, curcumin increased IFN-γ (35.12 ± 5.05 pg/L vs 22.45 ± 4.50 pg/L, *P* < .001) and reduced TGF-β1 (1423.67 ± 290.35 pg/L vs 2750.45 ± 360.25 pg/L) and TNF-α (15.75 ± 5.43 pg/L vs 32.10 ± 7.25 pg/L, *P* < .001). In conclusion, curcumin outperformed triamcinolone in alleviating OSF symptoms, likely via dual regulation of IFN-γ (upregulation) and TGF-β1/TNF-α (downregulation), offering a promising therapeutic alternative.

## 1. Introduction

Oral submucous fibrosis (OSF) is a chronic, progressive condition driven by persistent inflammation, and is widely recognized as a precancerous disorder.^[1–3]^ Its primary pathological features include excessive submucosal collagen deposition, reduced elastic fibers, epithelial atrophy, and progressively limited mouth opening.^[4,5]^ The prevalence of OSF is particularly high in South and Southeast Asia, with a strong association with areca nut chewing.^[6,7]^ In recent years, the incidence of OSF has also been rising in certain regions of China, in parallel with increased areca nut consumption. According to existing literature, the malignant transformation rate of OSF ranges from 7% to 13%, and the World Health Organization has classified it as an oral potentially malignant disorder with established oncogenic potential.^[8]^ The chronic and irreversible disease course not only impairs chewing, swallowing, and speech functions, but also imposes a lasting burden on patients’ psychological health and overall quality of life.^[9–11]^

Although the exact pathogenesis of OSF remains incompletely understood, current evidence suggests that chronic inflammation, aberrant fibroblast activation, and dysregulated extracellular matrix metabolism are central contributors to its development.^[3,12]^ Notably, abnormal expression levels of inflammatory cytokines such as IFN-γ, TGF-β1, and TNF-α have been consistently observed in OSF patients, suggesting a mechanistic link between immune dysregulation and tissue fibrosis.^[13,14]^ Consequently, targeting the dynamic regulation of these cytokines has emerged as a critical direction in OSF research.

At present, clinical management of OSF primarily relies on local administration of corticosteroids, hyaluronic acid, and interferon, as well as systemic use of antioxidants, iron supplements, and multivitamins.^[3,15]^ However, conventional treatments are frequently limited by transient efficacy, high relapse rates, suboptimal long-term compliance, and the risk of adverse effects.^[16]^ For instance, corticosteroids can effectively reduce inflammation and mucosal rigidity, and may partially improve mouth opening, but prolonged use can lead to mucosal atrophy and increased susceptibility to fungal infections, limiting their utility in long-term management.^[17]^ Moreover, a lack of high-quality, prospective, large-scale clinical studies continues to hinder the establishment of robust, evidence-based treatment guidelines.

Curcumin, a natural polyphenolic compound extracted from the rhizome of *Curcuma longa*, has received growing attention in recent years due to its anti-inflammatory, antioxidant, antifibrotic, and immunomodulatory properties.^[18–20]^ Preclinical and in vivo studies have shown that curcumin can inhibit the TGF-β1/Smad signaling pathway, reduce collagen deposition, downregulate TNF-α expression, and modulate the Th1/Th2 cytokine balance, thereby exerting therapeutic benefits in fibrotic and mucosal disease models.^[19,21–23]^ Nevertheless, clinical evidence supporting the use of curcumin in OSF remains limited.

In this context, the present study employed a retrospective cohort design to systematically evaluate the clinical efficacy of curcumin in the treatment of OSF, and to explore its effects on serum inflammatory cytokine levels (IFN-γ, TGF-β1, and TNF-α). The objective was to provide preliminary clinical evidence for curcumin’s therapeutic potential in OSF and to elucidate its possible immuno-fibrotic regulatory mechanisms.

## 2. Materials and methods

### 2.1. Study design

This study adopted a retrospective cohort design to evaluate the clinical efficacy of curcumin in the treatment of OSF, and to assess its effect on inflammatory cytokines including IFN-γ, TGF-β1, and TNF-α. The study protocol was approved by the Affiliated Hospital of Hebei University of Engineering ethics committee and adhered strictly to ethical standards and privacy protection regulations. No active clinical intervention was conducted, and all data were obtained from patients’ electronic medical records and historical treatment documentation. All collected data were anonymized and de-identified, used solely for academic analysis and publication purposes, with no other utilization, thereby ensuring full protection of patient privacy.

### 2.2. Study population

Patients diagnosed with OSF and treated at the Department of Stomatology, Affiliated Hospital of Hebei University of Engineering between June 2023 and September 2024 were considered eligible. Relevant clinical information, including diagnostic reports, laboratory results, and follow-up data, was systematically collected by reviewing outpatient, emergency, and inpatient records. Based on the therapeutic regimen received, patients were categorized into two groups: the observation group received oral curcumin tablets, while the control group received triamcinolone acetonide injections. After applying the inclusion and exclusion criteria, a total of 110 eligible patients were enrolled, including 54 in the observation group and 56 in the control group.

Inclusion criteria: diagnosed with OSF based on established clinical diagnostic criteria;^[24]^ documented history of areca nut chewing and willingness to abstain from further use, with regular follow-up compliance; presence of varying degrees of restricted mouth opening; first-time diagnosis with no prior systemic treatment; age between 18 and 50 years.

Exclusion criteria: presence of severe systemic diseases (e.g., cardiovascular, hepatic, or renal dysfunction); concurrent oral mucosal disorders such as leukoplakia, erythroplakia, or malignancy; periodontal probing depth ≥ 4 mm, indicating moderate to severe periodontal disease; history of allergy to study medications (e.g., triamcinolone or curcumin); pregnant or lactating women; radiographic evidence of alveolar bone resorption with confirmed periodontitis.

### 2.3. Treatment protocols

In the observation group, patients received oral curcumin tablets (GNC, 60 tablets per bottle), administered at a dose of 300 mg per time, 1 to 2 times daily. In the control group, patients were treated with a mixture of triamcinolone acetonide injection (10 mg/mL, 8 mL) and lidocaine injection (20 mg/mL), injected bilaterally at the base of the lesion sites, once weekly. Both treatment regimens were continued for a total of 4 weeks.

### 2.4. Outcome measures

Patients were evaluated at baseline, week 2, and week 4 (end of treatment). The primary outcome measures included:

(1) Clinical efficacy assessment:
  ① Markedly effective: resolution of mucosal stiffness and burning pain, with an increase in mouth opening ≥ 1 cm.
  ② Effective: significant relief in mucosal stiffness and pain, with an increase in mouth opening < 1 cm.
  ③ Ineffective: no improvement or worsening of mouth opening or clinical symptoms.

$$\begin{array}{l} \begin{array}{lll} & Totalef\;f\;icacyrate=(numberofmarkedlyef\;fective+ef\;fectivecases)\; & /totalnumberofcases\times 100\%.\; \end{array} \end{array}$$

(2) Clinical symptom scores:

Evaluated parameters included pain severity, mucosal lesion area, and mouth opening. Pain was assessed using the VAS, where higher scores indicate greater pain intensity. Mouth opening was defined as the distance between the incisal edges of the upper and lower central incisors.

(3) Serum levels of IFN-γ, TGF-β1, and TNF-α:

Fasting venous blood samples were collected, and serum concentrations of IFN-γ, TGF-β1, and TNF-α were measured using enzyme-linked immunosorbent assay kits.

### 2.5. Statistical analysis

All statistical analyses were performed using SPSS version 23.0 (International Business Machines Corporation [IBM], Armonk). Continuous variables were expressed as mean ± standard deviation (X ± SD), while categorical variables were presented as frequencies (n) and percentages (%). Intergroup comparisons of continuous variables were conducted using the *t* test based on data distribution, and differences in categorical variables were analyzed using the chi-square test. In addition, multivariate logistic regression analysis was performed to adjust for potential confounding factors, including age, sex, and disease duration, when comparing clinical efficacy outcomes between groups. A *P*-value < .05 was considered statistically significant.

## 3. Results

### 3.1. Baseline characteristics

A total of 110 patients diagnosed with OSF were included in this study, with 54 in the observation group and 56 in the control group. There were no statistically significant differences between groups in age (observation: 37.53 ± 7.56 years; control: 38.55 ± 8.17 years; *t* = –0.680, *P* = .498), gender distribution (male: 37 [68.52%] vs 37 [66.07%]; female: 17 [31.48%] vs 19 [33.93%]; *χ*² = 0.075, *P* = .785), or disease duration (observation: 3.18 ± 1.22 years; control: 2.94 ± 1.12 years; *t* = 1.074, *P* = .285), suggesting baseline comparability (Table 1).

**Table 1:** Baseline demographic and clinical characteristics of the observation and control groups (X ± SD, n/%).

Variable	Observation group (n = 54)	Control group (n = 56)	t/χ²	*P*
Age (yr)	37.53 ± 7.56	38.55 ± 8.17	–0.680	.498
Gender			0.075	.785
Male	37 (68.52%)	37 (66.07%)		
Female	17 (31.48%)	19 (33.93%)		
Disease duration (yr)	3.18 ± 1.22	2.94 ± 1.12	1.074	.285

### 3.2. Clinical efficacy

At the end of treatment, the total effective rate was significantly higher in the observation group (88.89%) than in the control group (73.21%) (*χ*² = 4.373, *P* = .037). In the observation group, 22 cases (40.74%) were markedly effective, 26 (48.15%) were effective, and 6 (11.11%) were ineffective. In the control group, 18 (32.14%) were markedly effective, 23 (41.07%) effective, and 15 (26.79%) ineffective (Table 2).

**Table 2:** Comparison of clinical efficacy between the observation and control groups (n/%).

Group	Markedly effective	Effective	Ineffective	Total efficacy rate
Observation group (n = 54)	22 (40.74%)	26 (48.15%)	6 (11.11%)	48 (88.89%)
Control group (n = 56)	18 (32.14%)	23 (41.07%)	15 (26.79%)	41 (73.21%)
χ²				4.373
*P*				.037

### 3.3. Pain assessment

There was no significant difference in baseline VAS pain scores (observation: 5.72 ± 0.82; control: 5.56 ± 1.01; *t* = 0.914, *P* = .363). After 2 weeks, the VAS score in the observation group decreased to 4.61 ± 0.63, significantly lower than that in the control group (4.95 ± 0.70; *t* = –2.680, *P* = .009). After 4 weeks, VAS further declined to 2.25 ± 0.52 in the observation group and 2.86 ± 0.43 in the control group, with a significant intergroup difference (*t* = –6.692, *P* < .001) (Table 3).

**Table 3:** Comparison of burning sensation (VAS) before and after treatment (X ± SD).

Time point	Observation group (n = 54)	Control group (n = 56)	*t*	*P*
Baseline	5.72 ± 0.82	5.56 ± 1.01	0.914	.363
Week 2	4.61 ± 0.63*	4.95 ± 0.70*	–2.680	.009
Week 4	2.25 ± 0.52†	2.86 ± 0.43†	–6.692	<.001

* Compared with values before treatment within the same group, *P* < .05.

† Compared with values after treatment within the same group, *P* < .05.

### 3.4. Mouth opening

Baseline mouth opening were similar (observation: 28.75 ± 5.43 mm; control: 29.01 ± 5.62 mm; *t* = –0.247, *P* = .806). At 2 weeks, both groups showed improvement (*P* < .05), with the observation group at 33.62 ± 3.64 mm and the control group at 32.23 ± 3.25 mm (*t* = 2.110, *P* = .037). By week 4, mouth opening increased to 36.88 ± 2.07 mm and 35.67 ± 2.71 mm, respectively, with a continued significant difference (*t* = 2.638, *P* = .011) (Table 4).

**Table 4:** Comparison of mouth opening (mm) before and after treatment (X ± SD).

Time point	Observation group (n = 54)	Control group (n = 56)	*t*	*P*
Baseline	28.75 ± 5.43	29.01 ± 5.62	–0.247	.806
Week 2	33.62 ± 3.64*	32.23 ± 3.25*	2.11	.037
Week 4	36.88 ± 2.07†	35.67 ± 2.71†	2.638	.011

* Compared with values before treatment within the same group, *P* < .05.

† Compared with values after treatment within the same group, *P* < .05.

### 3.5. Mucosal lesion area

At baseline, lesion areas were comparable (observation: 8.57 ± 1.23 cm²; control: 8.51 ± 1.20 cm²; *t* = 0.259, *P* = .797). After 2 weeks, the area reduced to 6.57 ± 0.67 cm² in the observation group and 7.04 ± 0.52 cm² in the control group (*t* = –4.098, *P* < .001). At week 4, the area further reduced to 4.63 ± 0.54 cm² and 5.61 ± 0.69 cm², respectively, with a statistically significant difference (*t* = –8.315, *P* < .001) (Table 5).

**Table 5:** Comparison of mucosal lesion area (cm²) before and after treatment (X ± SD).

Time point	Observation group (n = 54)	Control group (n = 56)	*t*	*P*
Baseline	8.57 ± 1.23	8.51 ± 1.20	0.259	.797
Week 2	6.57 ± 0.67*	7.04 ± 0.52*	–4.098	<.001
Week 4	4.63 ± 0.54†	5.61 ± 0.69†	–8.315	<.001

* Compared with values before treatment within the same group, *P* < .05.

† Compared with values after treatment within the same group, *P* < .05.

### 3.6. Inflammatory cytokine levels (IFN-γ, TGF-β1, and TNF-α)

There were no significant baseline differences in IFN-γ (observation: 12.34 ± 3.21 pg/L; control: 12.10 ± 3.45 pg/L; *t* = 0.311, *P* = .757), TGF-β1 (observation: 3350.45 ± 450.12 pg/L; control: 3380.50 ± 470.95 pg/L; *t* = –0.310, *P* = .757), or TNF-α (observation: 45.80 ± 10.21 pg/L; control: 46.50 ± 11.05 pg/L; *t* = –0.440, *P* = .661). At week 2, IFN-γ levels increased significantly in both groups (*P* < .05), with a greater increase in the observation group (24.56 ± 4.10 pg/L) compared to the control (16.78 ± 3.91 pg/L; *t* = 8.124, *P* < .001). By week 4, levels rose further to 35.12 ± 5.05 pg/L in the observation group and 22.45 ± 4.50 pg/L in the control group (*t* = 11.758, *P* < .001) (Table 6).

**Table 6:** Comparison of IFN-γ, TGF-β1, and TNF-α levels before and after treatment (pg/L, X ± SD).

Time point	Observation group (n = 54)	Control group (n = 56)	*t*	*P*
IFN-γ				
Baseline	12.34 ± 3.21	12.10 ± 3.45	0.311	.757
Week 2	24.56 ± 4.10*	16.78 ± 3.91*	8.124	<.001
Week 4	35.12 ± 5.05†	22.45 ± 4.50†	11.758	<.001
TGF-β1				
Baseline	3 350.45 ± 450.12	3 380.50 ± 470.95	–0.310	.757
Week 2	2 567.23 ± 350.45*	2 987.50 ± 400.60*	–7.123	<.001
Week 4	1 423.67 ± 290.35†	2 750.45 ± 360.25†	–14.256	<.001
TNF-α				
Baseline	45.80 ± 10.21	46.50 ± 11.05	–0.440	.661
Week 2	28.50 ± 8.34*	39.20 ± 9.10*	–6.232	<.001
Week 4	15.75 ± 5.43†	32.10 ± 7.25†	–11.457	<.001

* Compared with values before treatment within the same group, *P* < .05.

† Compared with values after treatment within the same group, *P* < .05.

TGF-β1 levels declined significantly by week 2 (observation: 2567.23 ± 350.45 pg/L; control: 2987.50 ± 400.60 pg/L; *t* = –7.123, *P* < .001), with a further decrease by week 4 (1423.67 ± 290.35 pg/L vs 2750.45 ± 360.25 pg/L; *t* = –14.256, *P* < .001).

Similarly, TNF-α levels declined after 2 weeks (observation: 28.50 ± 8.34 pg/L; control: 39.20 ± 9.10 pg/L; *t* = –6.232, *P* < .001) and continued to decline by week 4 (15.75 ± 5.43 pg/L vs 32.10 ± 7.25 pg/L; *t* = –11.457, *P* < .001).

## 4. Discussion

This retrospective cohort study demonstrated that oral administration of curcumin yields significant clinical benefits in the treatment of OSF. Compared with conventional therapy, the curcumin group showed a more rapid and sustained improvement in total efficacy rate, pain relief, mouth opening, and reduction in mucosal lesion area. Notably, curcumin treatment significantly elevated peripheral blood IFN-γ levels while reducing TGF-β1 and TNF-α levels, suggesting that curcumin may modulate inflammatory cytokine balance and facilitate a shift from a profibrotic to an antifibrotic state.

By the 2nd week, patients in the observation group had already shown a substantial reduction in pain and an improvement in mouth opening, suggesting that curcumin can rapidly relieve OSF-related discomfort and functional limitations. The pathological hallmarks of OSF include fibroblast activation, excessive collagen deposition, and persistent inflammation, which commonly manifest as burning sensations and trismus.^[2]^ These findings indicate that curcumin provides a more rapid and effective alleviation of OSF-related symptoms. Furthermore, regarding objective mucosal lesion changes, the lesion area in the observation group decreased from 8.57 cm² at baseline to 6.57 cm² at week 2, compared with 7.04 cm² in the control group (*P* < .001); at week 4, it further reduced to 4.63 cm² versus 5.61 cm² in the control group (*P* < .001). The more rapid decrease in lesion area indicates that curcumin may facilitate the process of epithelial repair.^[25]^ In OSF pathophysiology, submucosal fibrotic bands increase mucosal tension and impair surface healing.^[26]^ The therapeutic effects of curcumin are likely mediated through the suppression of fibrosis and enhancement of the local microenvironment, thereby promoting epithelial regeneration.^[27]^

Importantly, the dynamic changes in cytokines provide mechanistic insights into curcumin’s therapeutic effect. IFN-γ in the observation group rose from 12.34 pg/L at baseline to 24.56 pg/L at week 2, compared with 16.78 pg/L in the control group (*P* < .001), and further to 35.12 pg/L vs 22.45 pg/L at week 4 (*P* < .001). Conversely, TGF-β1 and TNF-α were significantly lower in the observation group at both time points. IFN-γ, a key Th1 cytokine, promotes M1 macrophage polarization and suppresses fibrosis; reduced TGF-β1 indicates inhibition of fibroblast activation and collagen synthesis, while lowered TNF-α suggests attenuation of local inflammation. This pattern (characterized by increased IFN-γ levels and decreased TGF-β1 and TNF-α levels) is consistent with the established anti-inflammatory and antifibrotic properties of curcumin.^[18,19]^

In clinical practice, the therapeutic application of curcumin is influenced by several factors. One notable limitation is its inherently low oral bioavailability, largely due to poor aqueous solubility, rapid metabolism, and systemic elimination, which may restrict the extent of its therapeutic effects. Various formulation strategies, including nanoparticle encapsulation, liposomal delivery systems, and the use of bioenhancers such as piperine, have been proposed to overcome this barrier and warrant further investigation in OSF-specific contexts. Additionally, patient compliance may vary depending on dosage form, frequency of administration, and treatment duration. Oral curcumin supplementation is generally well tolerated; however, long-term adherence could be affected by perceived slow onset of benefits, cost, and availability. These factors, along with other potential clinical application obstacles, should be carefully addressed in future studies to optimize curcumin’s therapeutic potential for OSF.

Nevertheless, this study has several limitations. First, being retrospective, it is inherently susceptible to selection and information bias, despite no significant baseline differences between groups. Second, the 4-week follow-up period limits assessment of long-term efficacy, maintenance effects, and potential adverse events. Although no adverse events, including gastrointestinal discomfort potentially associated with oral curcumin, were observed in either group during the study period, the short duration of follow-up may have underestimated the risk of late-onset or cumulative side effects. This limitation reduces the ability to comprehensively evaluate the safety profile of curcumin and may affect the strength of its clinical translatability. Third, outcome measures relied primarily on clinical scoring and serum cytokine levels, without histopathological or imaging-based assessment of submucosal fibrosis, precluding direct visualization of collagen changes. Future prospective, randomized controlled trials with extended follow-up, multimodal assessment (e.g., biopsy, imaging, bioavailability profiling), and optimized formulations (e.g., nano-curcumin) would better elucidate the long-term clinical value of curcumin.

In summary, this study based on retrospective data reveals that curcumin, when used alongside standard care, significantly improves subjective symptoms and functional outcomes in OSF within a short-term period. These benefits appear to be mediated via modulation of IFN-γ, TGF-β1, and TNF-α levels, supporting its dual anti-inflammatory and antifibrotic mechanisms. These preliminary findings suggest that curcumin may serve as a safe and feasible adjunctive therapy, although further large-scale, prospective studies are required to confirm its long-term efficacy and safety. Future studies should investigate optimal formulations and dosing strategies under rigorous trial conditions and evaluate long-term effects using comprehensive and multimodal metrics.

## Author contributions

**Conceptualization:** Juanhua Chai, Liye Song.

**Data curation:** Yang Li, Liye Song.

**Formal analysis:** Yang Li, Liye Song.

**Funding acquisition:** Liye Song.

**Investigation:** Mengfei Yao, Liye Song.

**Methodology:** Lu Zhang, Liye Song.

**Visualization:** Yuxin Gu, Liye Song.

**Writing – original draft:** Juanhua Chai, Liye Song.

**Writing – review & editing:** Juanhua Chai, Liye Song.
